# Anticipated and Experienced Stigma After Testing Positive for SARS-CoV-2: A Qualitative Study

**DOI:** 10.1177/15248399221115063

**Published:** 2022-08-11

**Authors:** Shelley N. Facente, Mariah De Zuzuarregui, Darren Frank, Sarah Gomez-Aladino, Ariel Muñoz, Sabrina Williamson, Emily Wang, Lauren A. Hunter, Laura Packel, Arthur Reingold

**Affiliations:** 1 University of California, Berkeley, CA, USA; 2 Facente Consulting, Richmond, CA, USA

**Keywords:** COVID-19, SARS-CoV-2, stigma, qualitative research, public health messaging

## Abstract

**Introduction:**

Stigma has inhibited public health practitioners’ influence during the COVID-19 pandemic. We explore the experienced and anticipated stigma of people affiliated with a large university in the United States, using the Health Stigma and Discrimination Framework.

**Methods:**

We conducted a qualitative secondary substudy of 20 people who tested SARS-CoV-2 positive and 10 who tested negative in the summer of 2020, selected from a study of 3,324 university students and employees.

**Findings:**

No participants reported anticipated stigmatization prior to testing positive. However, eight of 20 participants recounted stigma marking (being marked by COVID-19 diagnosis or membership in a “high-risk” group) or manifestations of stigma after testing positive, including feelings of guilt or shame, and concerns about being judged as selfish or irresponsible. Three described being denied services or social interactions as a result of having had COVID-19, long after their infectiousness ended. Participants noted that clear public health messaging must be paired with detailed scientific information, rather than leaving people to resort to non-experts to understand the science.

**Discussion:**

Public health messaging designed to mitigate spread of SARS-CoV-2 and protect the community may perpetuate stigma and exacerbate inequities. As a result, people may avoid testing or treatment, mistrust public health messaging, or even use risk-increasing behavior as coping mechanisms.

**Implications for Practice:**

Intentional use of language that promotes equity and deters discrimination must be high priority for any COVID-19-related public health messaging. Partnership with community leaders to co-create programs and disseminate messaging is a critical strategy for reducing stigma, especially for historically mistreated groups.

The COVID-19 pandemic is one of the most important public health crises of the past 100 years. Public health practitioners have attempted to change behavior (e.g., promote mask usage, encourage social distancing) and save lives by communicating best practices for risk-reducing behavior and policies to mitigate community spread; however, political, sociological, and psychological factors have posed barriers to public health promotion. One factor that has played a substantial role in public health practitioners’ influence during the COVID-19 pandemic has been stigma. Studies have begun to look at the experiences of stigma among people who have tested positive for SARS-CoV-2 in countries outside the United States (Chew et al., 2021; Iqbal et al., 2021; Owusu et al., 2021; Theano et al., 2020; Yuan et al., 2021). However, there has been relatively little investigation into the role of stigma in health behavior change in the United States, where the pandemic has been highly politicized and polarizing (Rabin & Dutra, 2021)—especially in the early days of the pandemic, when the societal response was still in a formative phase.

One web-based survey of 72 COVID-19 survivors across the United States reported that 51% had experienced stigma as a result of becoming infected with SARS-CoV-2 in 2020, including being avoided by friends or neighbors after recovery, being blamed by others for spreading the virus, and experiencing hostility from clinical staff when seeking care (Prioleau, 2021). The impact of stigma during infectious disease outbreaks has been well documented for Ebola, SARS, and polio (Bologna et al., 2021; Kelly et al., 2019; Lasalvia, 2020; Saeed et al., 2020). As a result, public health practitioners should be familiar with and sensitive to the need to reduce stigma when planning and implementing interventions. Contact tracing programs for partner elicitation and notification of exposure to syphilis and HIV illustrate great sensitivity to issues of privacy and de-stigmatization—even including re-branding HIV contact tracing as “partner services” to improve patient receptivity (Carter et al., 2016). Nonetheless, it can be difficult to balance effectively communicating public health messages while not stigmatizing people who continue to participate in behaviors that put them at increased risk of disease (Logie & Turan, 2020).

The Health Stigma and Discrimination Framework (HSDF; Figure 1) is one theoretical framework for understanding health-related stigma (Stangl et al., 2019). The first domain of the HSDF refers to drivers or facilitators of health-related stigma. Drivers are fear and confusion, such as fear of infection and societal stereotypes about people with the disease, while facilitators are cultural or social norms and structures, such as occupational safety standards, or health policies that exacerbate stigmatization of people who become infected. Drivers and facilitators influence the second domain, which is “stigma marking,” whereby stigma is attached to people based on their disease diagnosis or membership in a group thought to be at higher risk for the disease, such as people of certain races, sexual orientations, occupations, or economic classes. Once stigma is applied, a third domain applies: manifestations. Manifestations can include experiences of stigma (including being discriminated against or mistreated, as well as experiencing internalized or anticipated stigma) and stigma practices (such as being stereotyped or causing discriminatory attitudes). Manifestations of stigma lead to the fourth domain—outcomes—where access to or experience of health care services and passage of legislation or policies, for example, are affected by stigmatization. Both individual and structural outcomes are where the health and social impacts of stigmatization are realized, with incidence, mortality, and quality of life related to the disease then being unequally experienced throughout the population.

**Figure 1:**
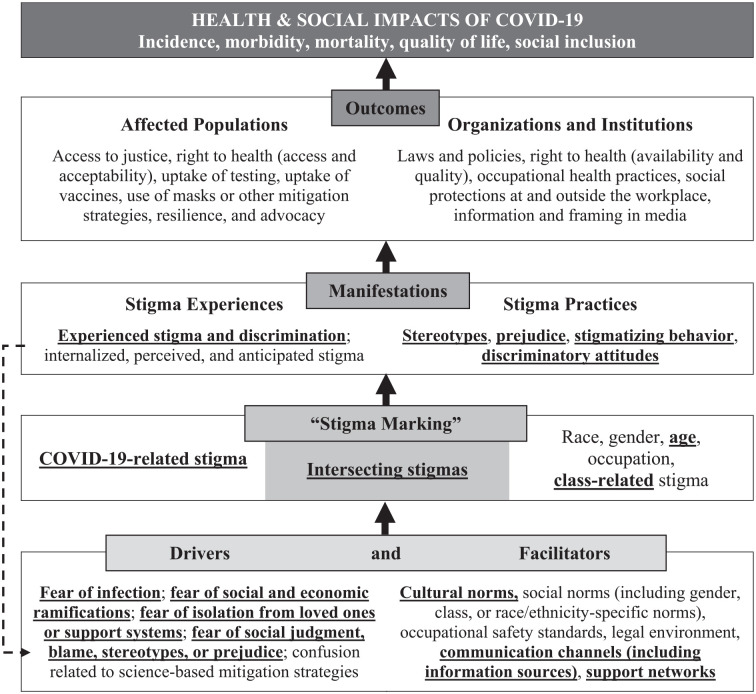
Stigma-Related Interview Themes (Bold, Underlined Text) Applied to the Health Stigma and Discrimination Framework Source: Adapted from Stangl et al. (2009).

Ransing and colleagues have applied the HSDF to COVID-19, looking at factors affecting the experiences of patients per the report of psychiatrists across 13 countries during the pandemic (Ransing et al., 2020). We were interested in the role COVID-19-related stigma played in the health care-seeking and disclosure behaviors of college students testing positive for SARS-CoV-2 during the summer of 2020, expanding the application of this HSDF-based model to COVID-19. To further explore the experienced or anticipated stigma of members of the University of California, Berkeley campus community during the early phase of the pandemic, we conducted a qualitative substudy within a larger Berkeley COVID-19 Safe Campus Initiative (BCSCI) (Packel et al., 2021).

## Method

The original BCSCI study included 3,324 students, faculty, and staff affiliated with the University of California, Berkeley, a large public university in Northern California with a campus community of approximately 45,000 students and 24,000 faculty and staff. Members of the community who were living in the Berkeley area during summer of 2020 were enrolled in the study beginning in June 2020 by completing a baseline survey and providing specimens for polymerase chain reaction (PCR) and antibody testing for SARS-CoV-2. PCR testing was available throughout the study upon request or based on reported symptoms, exposure, or random surveillance. In August 2020, participants were asked to complete an endline survey and provide another set of specimens for PCR and antibody testing (Packel et al., 2021).

After the endline survey closed, we recruited 30 participants of the BCSCI study to participate in a qualitative substudy. We first purposively sampled participants from a list of 60 university students and employees who tested positive during the BCSCI study. Participants were selected to ensure maximum variation based on sociodemographics (gender, race/ethnicity, and university position), and recruited via email. If they did not respond to the email invitation, up to two follow-up phone calls or text messages were made before considering them to have declined participation. Recruitment continued until a total of 20 had been enrolled, with the goal of enrolling enough participants having been diagnosed with COVID-19 to reach saturation of themes from the interviews. As participants with consistently negative SARS-CoV-2 results would only be able to speak about their fears of stigma related to potentially testing positive, and conjecture about actual impacts of diagnosis, we chose a 2:1 ratio of positives to negatives. Once the 20 positive participants were enrolled, 10 consistently negative participants were then randomly sampled from the main BCSCI participant list, matched 1:2 with those testing positive on campus role (i.e., student, essential worker, faculty, or staff), sex, age, race/ethnicity, time of main study enrollment, and residence in group housing, to improve comparability of interviewees in the two groups. These negative participants were recruited in a similar fashion to the 20 positive participants.

After indicating an interest in participating in the qualitative substudy, participants completed an electronic informed consent form via Research Electronic Data Capture (REDCap) (Harris et al., 2009) and were then scheduled to participate in an hour-long Zoom-based video interview (San Jose: Zoom Video Communications). Participants were emailed a $50 gift card at the close of the interview as compensation for their time. Transcripts were automatically generated through Zoom and hand-edited by the interviewer for accuracy, although on three occasions failure to automatically transcribe led to the audio file being transcribed manually or via Rev.com. Transcripts were coded in Dedoose version 8.3.45 (Los Angeles: SocioCultural Research Consultants). Each transcript was randomly assigned to two team members, who independently coded transcripts using a shared codebook co-developed by interviewers in a group discussion after interviews were completed. After coding five interviews each, the research team discussed and revised the codebook, then recoded the initial transcripts, and completed the remainder of the coding. Coding discrepancies were resolved by team discussion and consensus. Fully coded transcripts were then analyzed using immersion and crystallization techniques (Borkan, 1999) to generate themes and identify exemplary quotations. This technique involves immersing oneself deeply in qualitative data (e.g., transcripts), then pausing for reflection and noting insights, then re-immersing in data, and then reflecting in a continuous cycle until themes and findings begin to crystallize.

## Findings

Response rates to invitations to participate in the qualitative substudy were 54% for those testing SARS-CoV-2 positive and 36% for those consistently testing SARS-CoV-2 negative. Most participants (87%) were students, with approximately two thirds of those undergraduates. The majority of participants were White (57%) or Latino/a/x/e (23%). Most participants were under age 21 (30%) or ages 21 to 29 (40%). More details on interviewee demographics can be found in Table 1.

**Table 1:** Demographics of Qualitative Sub-Study Participants

Category	*SARS-CoV-2 positive* *(n = 20)*	*SARS-CoV-2 negative* *(n = 10)*	*Total* *(N = 30)*
Cohort			
Student	18 (90%)	8 (80%)	26 (87%)
Undergraduate	11 (61%)	5 (63%)	16 (62%)
Graduate	7 (39%)	3 (37%)	10 (38%)
Essential worker, faculty, or staff	2 (10%)	2 (20%)	4 (13%)
Sex			
Male	6 (30%)	3 (30%)	9 (30%)
Female	14 (70%)	7 (70%)	21 (70%)
Race/ethnicity			
White	13 (65%)	4 (40%)	17 (57%)
Black/African American	1 (5%)	1 (10%)	2 (7%)
Latino/a/x/e	3 (15%)	4 (40%)	7 (23%)
Other	3 (15%)	1 (10%)	4 (13%)
Age			
<21	7 (35%)	2 (20%)	9 (30%)
21–29	8 (40%)	4 (40%)	12 (40%)
30–39	4 (20%)	4 (40%)	8 (27%)
40–49	1 (5%)	0 (0%)	1 (3%)
Group housing (students only)			
Yes	7 (39%)	3 (37%)	10 (38%)
No	11 (61%)	5 (63%)	16 (62%)

All participants were first asked about the worries they had about possibly learning they had COVID-19 (prior to testing positive, in the case of people who had ultimately tested positive). Notably, not a single participant described anticipated stigma as a concern held prior to testing positive. Instead, there were three main themes to these responses, including concerns about: (a) the possibility of infecting others (*n* = 7), (b) the possibility of experiencing severe symptoms or long-lasting health effects (*n* = 4), and (c) logistical challenges or other negative impacts of having to isolate after testing positive (*n* = 3). No patterns in responses were discernible by gender, race/ethnicity, campus role, residence in group housing, or COVID-19 status.

The 20 participants who had tested positive were then asked about the main worries they had *after* testing positive. Fourteen of the 20 people spoke about substantial concerns related to infecting others, with seven people naming specific family members who lived with them and/or were particularly vulnerable to severe infection. Twelve participants spoke about their fears related to their own health (e.g., severe disease, long COVID, and/or the possibility of long-term effects that would not become apparent for a long time). Ten spoke about the emotional stress they experienced due to isolation, with three describing logistical challenges (one who lived in a shared studio with no place to isolate; one who experienced financial hardship related to his wife needing to quarantine and miss work; and one who was a single parent), and eight describing the loneliness and anxiety they felt as a result of isolation from others. While not described by participants as stigma-related, each of these themes could be characterized as “drivers” of stigma under the HSDF (Figure 1).

There were also new concerns people experienced after testing positive, which they had not previously anticipated. One major concern was related to addressing others’ worries about their SARS-CoV-2 infection, as well as managing the ethics and drawbacks of disclosure. As one student participant explained,

[I was] fielding a lot of concern and anxiety on the part of my advisor at school . . . I ended up deciding just to tell everyone in the community that I was communicating with . . . just for the purpose of them knowing how close it is, and to continue to be really careful and not, you know, make the mistake that I did. So I think maybe that helps some people understand that it’s serious.

Some participants worried about telling coworkers, as one student recalled,

I was doing an internship and I didn’t tell anyone . . . because I feel like there’s . . . some stigma around it, like you’re a kid and you’re doing something irresponsible.

Six of the 20 participants specifically mentioned concerns about disclosing to parents or other immediate family members (e.g., spouse, grandmother), because of not wanting to worry them, or not wanting the additional responsibility of caring for them if they were worried.

Two student participants explicitly recounted worries that they would be perceived as irresponsible once (or if) they disclosed that they had tested positive. One said,

I am still just worried about what people could think about me, like [getting COVID-19] makes me . . . not responsible. I think the thing I worry about is people seeing me in a different way . . . or asking how I got it. It wasn’t like I just got it in a grocery store.

Another noted,

The kind of social stigma of it [worried me] . . . being in the small category of people in this community that tested positive definitely felt weird, like what did I do wrong that other people aren’t doing? Or there’s this kind of weird judgment of myself. Like, I must have made a mistake . . . clearly I was feeling bad about some different decisions I made, even though I didn’t know what they were.

Many participants spoke directly about manifestations of stigma that were realized after testing positive, with five of the 20 participants specifically using the word “stigma” to describe their experience. One explained,

I guess my expectation was, “Let’s take what the doctors say and let that be the guide.” So if the doctors say I need to quarantine and I do my quarantine and then they tell me I’m released into society, then people should take that for, “The doctor is the medical professional and, like, what you just read on Twitter is meaningless.” And that was not the case, you know.

Another three participants didn’t use the word “stigma,” but described experiences in which they were upset about how they had been treated after testing positive. One remembered, “I just felt like people were judging me like I had a plague or something.” Another was refused veterinary care for his dog because he had tested positive for COVID-19, and after his dog-walker told other clients he had tested positive, “they were like, yeah, we don’t want you to go to that house anymore.” Another shared,

I have a group chat . . . with my cousins on my mom’s side, and let them all know I had COVID, but my mom found out somehow that I posted it to everyone and she was really upset with me for sharing that information, and so then I had to apologize to the cousin group for sharing that information.

Requests were sometimes couched in an “abundance of caution,” but the requests were not evidence-based and resulted in the person feeling unfairly and illogically held to a different standard than those who had not disclosed a positive result. One participant was asked to show a negative antibody test result to join a group camping trip, which was not only stigmatizing but put the burden of education on the person being stigmatized, since an antibody result does not indicate infectiousness:

Some people wanted to go camping . . . And I had gotten invited to that. But then someone asked me—this is like two months after everything happened—they asked me if I could provide a negative antibody test [result]. . . . I mean, I already told them I’d tested negative since then. With the rate of asymptomatic transmission, if they wanted to be totally safe they they would have been asking everybody for a negative test result instead of just asking me . . . I tried to be understanding that this is a really challenging and triggering time for people. But that just seemed like a really uninformed way to go about it.

More participants of color reported experiencing stigma as a result of their SARS-CoV-2 infection: 13 (65%) of the participants testing SARS-CoV-2 positive were White and seven (35%) were people of color; however, among the eight who described manifestations of stigma as a result of their SARS-CoV-2 infection, four (50%) were White and four (50%) were people of color. Those who experienced stigma also tended to be older (age 30–39) and not living in Greek or cooperative housing than those who participated overall.

## Discussion

Worldwide, violence and discrimination have been experienced by people related to COVID-19. This is especially true for Asians in the United States, where there were deliberate attempts by some to brand SARS-CoV-2 as a “Chinese virus” (Hswen et al., 2021). The impacts of these manifestations of stigma during an infectious disease outbreak are multifaceted. Many of the participants in our study experienced shame, guilt, and social isolation that intensified the negative effects of the physical isolation imposed to prevent spread. People who feel ill may be more likely to avoid testing or even potentially life-saving treatment, for fear of being stigmatized if confirmed positive. Still others may cope with the stress of their COVID-19 risk or their embarrassment about past risk decisions by indulging in behaviors that in turn increase their future infection risk (Sotgiu & Dobler, 2020).

Notably, none of our participants described experiences of stigma as a result of their interaction with the public health system. However, even when public health and medical practitioners are actively working to destigmatize COVID-19 infection and ensure equitable treatment of *all* patients, others may misinterpret or misapply health public health messaging and use it to stigmatize those around them—as was evident in the camping and dog-walking stories from our participants. Per the HSDF, the potential outcomes of stigma can include reduced access to health care and increased occupational or social exposures for certain groups of individuals, exacerbating inequities that have been on display throughout the COVID-19 pandemic. These impacts could be counteracted with more clear and concrete public health messaging, to reduce the likelihood that those who are more vulnerable to these societal shifts are not further stigmatized by people who misguidedly perceive themselves to be enforcing public health messaging they do not fully understand.

The “novel” nature of the SARS-CoV-2 virus, its demonstrated ability to spread to others during asymptomatic or presymptomatic periods, and the danger posed by strangers coming “too close,” given recommended social distancing, have all helped increase the potential for stigmatization during this pandemic (Rzymski et al., 2021). The impacts of COVID-19-related stigma may be felt even more acutely by people of color than by White people in the United States, whether born in the United States or foreign-born (Egelko et al., 2020)—as was seen among our participants, where people of color disproportionately reported experiences of COVID-19-related stigma. This is due to intersecting stigma and increased “stigma marking” per the HSDF, alongside structural racism, which puts people of color at greater risk for negative health and economic outcomes due to COVID-19.

This qualitative study had several limitations. The small number of participants in this study limits generalizability. There was also likely selection bias with regard to participants who agreed to take part. While 61.7% of the 60 positive cases from the larger BCSCI study were invited to participate, it is likely that those who responded to requests to enroll in the substudy were not representative of the larger UC Berkeley community, other college campuses, or the larger society. Finally, this study took place early in the pandemic, and findings may not apply to the drivers, facilitators, and manifestations of COVID-19-related stigma at later pandemic phases.

## Implications for Practice

More than 2 years into the COVID-19 pandemic, it is clear that while many say we are “all in the same boat” regarding susceptibility to this novel virus, the reality is our experiences have been quite different and unequal, with some of us weathering this storm on yachts and others in dinghies. Health behaviors related to COVID-19 have become increasingly politicized in the United States (Byrd & Białek, 2021; Tan et al., 2021), with misinformation and local community culture contributing to stigmatization of people diagnosed with COVID-19. Within this greater context, public health practitioners know well that the way we talk about risk for communicable diseases matters: Ebola (Kelly et al., 2019), HIV (Armoon et al., 2021), and other diseases have taught us that stigmatizing health messaging can directly result in poorer health outcomes for those who are disproportionately affected. Clear, transparent, and honest health communication and use of language that promotes equity and deters discrimination must be high priority for any public health messaging about COVID-19 (Hargreaves & Logie, 2020). Specifically, providing clear and unambiguous guidance concerning ways to reduce risk must be paired with:

(a) Recognition of the lack of control some people have over their environment (Gubrium & Gubrium, 2021) (e.g., directives to “stay at home” are not only unrealistic, but potentially stigmatizing to people who are unhoused; directives to “shelter in place” may be more appropriate);
(b) Expert information about the disease and its spread, communicated in lay language but with an expectation that listeners are capable of understanding detailed scientific information (rather than filling in their gaps in knowledge via social media or through word of mouth) (van Daalen et al., 2021). Importantly, there is a delicate balance to be struck between rendering racial/ethnic or other inequities invisible and inadvertently assigning blame by over-emphasizing disease burden by ethnicity, travel history, age, or pre-existing medical conditions (Gronholm et al., 2021);
(c) Repeated reminders that scientific knowledge is continually evolving during this pandemic, and indeed the virus itself is evolving—thus, we all must be prepared to shift mitigation strategies as more is learned about which interventions work and which are unnecessary. These changes in public health strategies are, in fact, part of our collective effort to address the pandemic sensibly, not a sign of ignorance or willful neglect on the part of public health officials. Public health practitioners should encourage a sense of collective responsibility and a need for a social justice approach to COVID-19, not an individualistic philosophy that will increase inequitable outcomes and further raise stigma levels.

In communities that have developed mistrust of the government or public health institutions as a result of historical and current mistreatment and marginalization, additional strategies are necessary to address COVID-19. Lessons learned from other diseases have shown the value of leveraging community leaders and other trusted partners within community networks to co-create programs and disseminate guidance or messaging (Bologna et al., 2021). This work should not be done by “assigning” tasks to community leaders or shifting responsibility for communication to these partners; instead, members of the community should be considered true allies in shaping programs and crafting scientifically sound messages in a way that will resonate with community members.

Regardless of their relationship to the public health field, all people will benefit from clear and non-stigmatizing communications about COVID-19. The participants in this study provided important insights into the role stigma can play in the experience of testing positive during a politically and socially charged pandemic, with important implications for COVID-19-related health communication and policy development by public health practitioners.
